# The Transition of Poised RNA Polymerase II to an Actively Elongating State Is a “Complex” Affair

**DOI:** 10.4061/2011/206290

**Published:** 2011-10-12

**Authors:** Marie N. Yearling, Catherine A. Radebaugh, Laurie A. Stargell

**Affiliations:** Biochemistry and Molecular Biology, Colorado State University, Fort Collins, CO 80523-1870, USA

## Abstract

The initial discovery of the occupancy of RNA polymerase II at certain genes *prior* to their transcriptional activation occurred a quarter century ago in *Drosophila*. The preloading of these poised complexes in this inactive state is now apparent in many different organisms across the evolutionary spectrum and occurs at a broad and diverse set of genes. In this paper, we discuss the genetic and biochemical efforts in *S. cerevisiae* to describe the conversion of these poised transcription complexes to the active state for productive elongation. The accumulated evidence demonstrates that a multitude of coactivators and chromatin remodeling complexes are essential for this transition.

## 1. Introduction

RNA Polymerase II (RNAPII) is a 12-subunit enzyme that binds promoter DNA and catalyzes the synthesis of messenger RNA in eukaryotes. Although the recruitment of RNAPII to a promoter is necessary for productive gene expression, it is not sufficient in many cases. Early studies in *Drosophila* [1] and more recent genome-wide analyses in both flies and humans have revealed that thousands of genes contain poised RNAPII at their promoters [2–5]. These poised promoters allow for rapid and synchronous activation, thereby providing the precise timing of gene expression critical for developmental processes [6, 7]. Indeed, postrecruitment events necessary to convert RNAPII into a productively elongating form are increasingly considered general regulatory features of transcription in higher eukaryotes [8–10]. 

In yeast cultured to stationary phase, approximately 40% of the genes in the genome show association of RNAPII in their inactive state [11]. These polymerases are thought to be poised for rapid and concerted activation upon transition to more opportunistic growth conditions. In actively growing yeast cultures, genome-wide studies indicate that partial but inactive PIC complexes are a widespread phenomenon across the genome [12] and a majority of bound RNAPII may be in an inactive state [11, 13]. Gene regulation at postrecruitment steps in *S. cerevisiae *is also supported by differences in 5′ to 3′ RNAPII occupancy and the frequent pausing of RNAPII within coding regions of genes [14, 15]. In addition, accumulation of inactive RNAPII within ribosomal protein genes [16] and at the promoter of the uninduced *CYC1* gene [17] provides further support for postrecruitment transcriptional regulation in yeast. Due to the genetic and biochemical amenability of the yeast system, studies of the transition of poised RNAPII to the active form have provided key insights into the sophisticated molecular requirements involved in this postrecruitment process.

## 2. The Yeast *CYC1* Gene: A Model for Postrecruitment Regulation via Poised RNAPII

**Figure 1:**
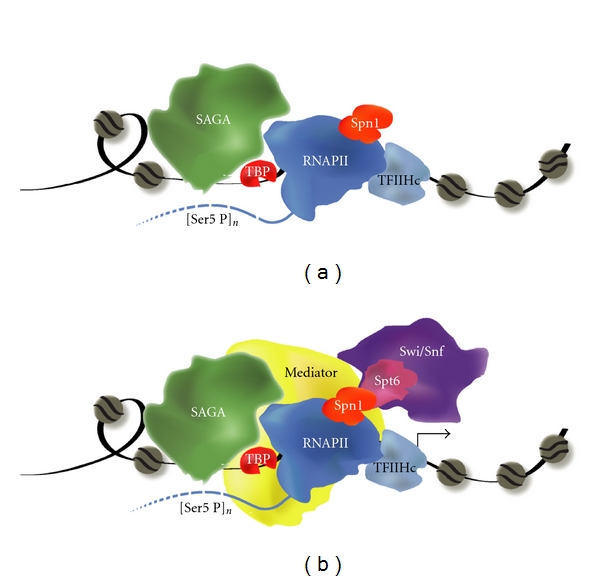
The poised *CYC1* promoter contains preloaded transcription components. (a) Prior to activation, the preloaded *CYC1* promoter contains TATA binding protein (TBP), RNA polymerase II (RNAPII), the core TFIIH complex (TFIIHc), Spt-Ada-GCN5 acetyltransferase (SAGA), and the transcription factor Spn1. The CTD, shown by the hashed line trailing RNAPII exhibits serine 5 phosphorylation potentially on multiple repeats (denoted by “*n*”). These components occupy the promoter prior to high levels of transcriptional output. (b) The occupancy of the preloaded factors is maintained under induced conditions, and Mediator, Spt6, and Swi/Snf are recruited, leading to an increase in transcriptional output (indicated by the arrow).

The yeast *CYC1* gene encodes iso-1-cytochrome c, a nuclear-encoded protein involved in the electron transport chain in the mitochondria [18]. In the presence of a fermentable carbon source (such as dextrose), *CYC1 *gene expression is extremely low [19, 20]. When cells are grown on a nonfermentable carbon source (such as lactate or ethanol), *CYC1 *is activated and transcript levels increase 10-fold. In contrast to the dramatic changes in transcriptional output, the occupancy of RNAPII [17, 21], as well as a number of other factors [22, 23], is maintained during the carbon source change (Figure 1). The *CYC1* promoter contains preloaded RNAPII, the general transcription factors TATA-binding protein (TBP) and TFIIH, the SAGA (Spt-Ada-Gcn5 acetyltransferase) complex, and Spn1, a highly conserved chromatin-associated transcription factor [22, 23]. Intriguingly, RNAPII is serine 5 phosphorylated on the C-terminal domain (CTD) of Rpb1 prior to activation [23]. The CTD is hypophosphorylated prior to initiation and typically becomes serine 5 hyperphosphorylated during the transition from initiation to elongation [24, 25]. The phosphorylation of the CTD at *CYC1* prior to activation is consistent with TFIIH occupancy, since TFIIH has CTD kinase as well as DNA unwinding activities [26–30]. Under inducing conditions for *CYC1,* a number of new factors are recruited to the promoter including the Mediator complex, and the chromatin regulatory factors the Swi/Snf complex and Spt6 [22]. This poised promoter could be advantageous in the native environment, allowing for rapid induction due to changing nutritional needs [31].

## 3. The Role of SAGA in the Inactive-to-Active Transition

The SAGA complex is a large multisubunit coactivator that facilitates gene expression at multiple steps within the transcription cycle [32, 33], including initiation [34–42] and more recently identified activities in the stimulation of elongation [33]. SAGA localization within gene coding regions [43–47] and elongation defects in SAGA deficient strains [46, 48, 49] demonstrate that the function of SAGA in transcription activation extends beyond the well-characterized activities of TBP delivery and posttranslational modifications of histones. It is unclear how the emerging functions in elongation pertain to the traditional roles of SAGA except at *CYC1,* where studies indicate that they appear to be functionally distinct. 

The poised *CYC1* promoter requires SAGA for the transition from a preloaded complex to an actively transcribing unit since deletion of SAGA-integrity subunits blocks activated transcription [22]. Several well-characterized functions of SAGA are not relevant to this transition. For example, SAGA contains a TBP interaction module essential for delivering TBP to certain promoters [34–38]. Since the preloaded promoter has both TBP and SAGA present under noninducing conditions, a functional connection between the two seemed likely. Surprisingly, although abolishing the SAGA complex results in loss of activated transcription, it does not alter TBP occupancy [22]. SAGA also has two known histone modifying enzymatic capabilities, a histone acetyltransferase (HAT) module responsible for acetylation events involved in facilitating active transcription [39–42] and a histone deubiquitinase (DUB) module known to aid in elongation [50, 51]. Yet, strains deficient for HAT activity or the DUB module are competent for activation [22].

In summary, the preloaded promoter is not dependent on the traditional well-characterized roles of SAGA, and yet SAGA integrity is required for the transition to an actively elongating complex after the recruitment of the PIC. These elongation activities may also be important at other genes, but difficult to observe because those genes require SAGA for recruitment of the general transcription machinery. The functions of numerous components within the SAGA complex remain to be elucidated and the preloaded promoter provides an excellent archetype for further investigations. Despite the necessity for SAGA function, SAGA is not sufficient for activation and another coactivator is critical for induction of the poised promoter.

## 4. Mediator-RNAPII Connections at *CYC1*

The Mediator complex is a large coactivator that is conserved from yeast to humans [52] and acts as an integrator of the transcription process, traditionally linking upstream signals from the activator with the general transcription machinery [53–57]. Mediator is essential for *CYC1* activation and is recruited after the transfer to inducing conditions [22]. Mediator is well characterized for its ability to recruit RNAPII to promoters [57, 58], although this function is unnecessary for *CYC1* since RNAPII is present at the poised promoter prior to activation. Mediator has also been shown to stimulate TFIIH-dependent phosphorylation of the CTD [57, 59]. However, as previously noted, serine 5 phosphorylation of the CTD at *CYC1* is observed prior to activation when Mediator is absent from the promoter, although subsequent rounds of transcription may be impacted. Mediator has also been shown to be involved in the isomerization of the PIC into a transcriptionally competent conformation [60]. This function fits well with the Mediator requirement for activation of the poised promoter as the subunits of Mediator essential for activating the poised promoter [22] are involved in interactions with RNAPII and the general transcription factors [54, 57, 61].

## 5. Chromatin Components with Critical Roles in the Transition to Active RNAPII

The transition of the poised promoter to its actively elongating form is highly dependent on a number of chromatin regulatory factors, including the Spn1/Spt6 [62, 63] complex and the Swi/Snf complex [23]. Notably, RNAPII and Spn1 occupy the poised promoter in the uninduced state, whereas Spt6 and Swi/Snf are recruited upon activation (Figure 1). Spn1 interacts with both RNAPII and Spt6 [23, 64–69], thereby linking the regulation of the poised promoter to the chromatin architecture. Spt6 is a histone chaperone that promotes reassembly of nucleosomes following passage of RNAPII [70–74], and Spn1 is an important regulator of the Spt6-nucleosome interaction [75]. In addition to a nucleosome maintenance role during elongation, Spt6 also has other chromatin-dependent [76] and chromatin-independent [77] roles in transcription. Importantly, the loss of Spn1 at *CYC1 *under noninducing conditions leads to a failure to recruit Spt6 under inducing conditions [23], consistent with their direct interaction [75, 78]. Mutations in either Spt6 [70, 79] or Spn1 [23] suppress mutant phenotypes associated with the loss of the Swi/Snf complex. 

Swi/Snf is an ATP-dependent chromatin remodeler that disassembles nucleosomes resulting in a loss of histones from DNA [80]. Swi/Snf is involved in remodeling at several recruitment-regulated promoters [81–84] as well as in coding regions [85, 86]. Suppressing mutations as observed for Spn1, Spt6, and Swi/Snf are typical indicators of factors that function in the same pathway and are physically connected [87, 88]. Specifically at *CYC1*, the absence of promoter binding by Spn1 results in constitutive recruitment of the Swi/Snf complex [23]. Thus, the binding of Spn1 blocks the recruitment of the Swi/Snf complex in the uninduced state and also serves as a platform for recruiting Spt6 during the activated state. An attractive model for the functions of these factors in proper *CYC1* expression is that, in order for the transition to an actively elongating state to occur, the Swi/Snf complex evicts nucleosomes and the Spn1/Spt6 complex reassembles them. Precisely how these activities are related to the poised RNAPII in the uninduced state remains to be investigated, but it is tempting to speculate that the interplay between RNAPII and the nucleosomal architecture contributes to the inactive state. The involvement of the chromatin context and inactive RNAPII complexes has also been observed at particular silent loci in the yeast genome [89]. 

## 6. Poising as a “Complex” Affair

**Figure 2:**
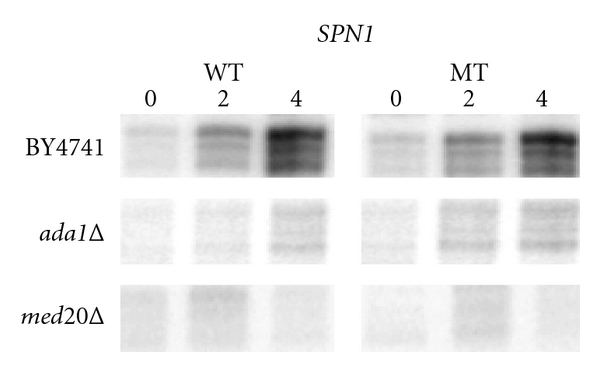
Mutating *SPN1*, which results in loss of Spn1 and constitutive recruitment of Swi/Snf to the promoter, does not bypass the requirement for SAGA or Mediator. *CYC1* transcript levels were analyzed before and two or four hours after induction in ethanol using an S1 nuclease protection assay [23] with RNA isolated from wild-type, *ada1*Δ or *med20*Δ strains. Each strain harbors either a wild-type (WT) or mutant (MT) form of *SPN1*. Similar results were obtained for other SAGA and Mediator deletion strains, including *gcn5*Δ*, spt7*Δ*, spt8*Δ*, spt20*Δ*, med5*Δ*, med15*Δ, and * med18*Δ (data not shown).

Several important questions remain. For example, what creates the poised polymerase in the first place? We have found that the occupancy of RNAPII at *CYC1* is an incredibly robust phenomenon: single deletion of dozens of different transcription factors and coactivator complex subunits has not resulted in RNAPII occupancy defects (data not shown and [22]). It could be that RNAPII preloading is an intrinsic property of the *CYC1* promoter and/or its nuclear and chromosomal context. In contrast to the resiliency of RNAPII occupancy, the transition to an actively elongating form is a highly demanding phenomenon, requiring the efforts of several prodigious and powerful transcription complexes: SAGA, Mediator, and Swi/Snf. Intriguingly, these complexes and their functions appear to work autonomously at *CYC1*. As shown previously [22], Mediator and SAGA occupancy are not dependent on each other, and mutations that result in constitutive occupancy of Swi/Snf do not bypass the need for SAGA or Mediator for activation of the preloaded promoter (Figure 2). As such, three distinct pathways are required to shift the polymerase into its active form. Further studies are needed to elucidate how each complex directly contributes to the transition from the poised to the active form. However, it is clear that in accordance with Newton's first law (a body at rest tends to stay at rest), these large macromolecular assemblies must provide the essential outside forces to initiate the process.

## 7. Perspectives

How related is the RNAPII poising observed in yeast to that in metazoans? Studies in flies and human cells have clearly established that in many cases the polymerase has started transcribing and is paused just downstream of the start site. In contrast, there is no evidence for initiated transcripts that are stalled in yeast [13, 90]. Whether the poised RNAPII in yeast is an evolutionary precursor to the more sophisticated version of paused RNAPII in metazoans is an open question. Nevertheless, it is intriguing that occupancy of SAGA [47, 91], a requirement for Mediator [53, 60, 92], a dependency on Spt6 [74], the involvement of Spn1 [73], and the chromatin architecture [93] play critical roles in pausing and/or postrecruitment transcriptional events in metazoan cells. Taken together, these results suggest that there are universal requirements for the activities of multiple complexes in the transition of RNAPII from a poised to an actively elongating state.
